# Identification and validation of suitable reference genes for quantitative real-time PCR gene expression analysis in pregnant human myometrium

**DOI:** 10.1007/s11033-020-06066-2

**Published:** 2021-01-01

**Authors:** Sarah Arrowsmith

**Affiliations:** Harris-Wellbeing Preterm Birth Research Centre, Institute of Life Course and Medical Sciences, University of Liverpool, Liverpool, UK

**Keywords:** qPCR, Expression stability, Reference genes, Myometrium, Oxytocin receptor, geNorm, NormFinder

## Abstract

Accurate quantification of quantitative PCR (qPCR) data requires a set of stable reference genes (RGs) for normalisation. Despite its importance to mechanistic studies, no evaluation of RG stability has been conducted for pregnant human myometrium. A systematic search of the literature was performed to identify the most used RGs in human myometrial gene expression studies. The stability of these genes, and others, was then evaluated using geNorm and NormFinder algorithms, in samples of myometrium from singleton or twin pregnancies (n = 7 per group) delivering at term or preterm. The most frequently cited RGs were *GAPDH, ACTB, B2M* and *18s.* There was strong agreement between algorithms on the most and least stable genes: Both indicated *CYC1, YWHAZ* and *ATP5B* were the most stably expressed. Despite being some of the most used RGs, *B2M*, *18s* and *ACTB* expression was least stable and was too variable for use as accurate normalisation factors. Pairwise variation analysis determined that the optimal number of RGs for accurate normalisation is two. Validation of the choice of RGs by comparing relative expression of oxytocin receptors (*OXTR)* using the least stable *18s* and *B2M*, with the most stable, *CYC1* and *YWHAZ*, erroneously demonstrated significantly increased *OXTR* expression in myometrium in singleton pregnancies compared to twins. This study demonstrates the importance of appropriate RG selection for accurate quantification of relative expression in pregnant human myometrium qPCR studies. For normalisation, the geometric mean of *CYC1* and *YWHAZ or ATP5B* is suggested. The use of *ACTB, 18s* and *B2M*, is not recommended.

**Supplementary Information:**

The online version contains supplementary material available at 10.1007/s11033-020-06066-2.

## Introduction

Real-time quantitative PCR (qPCR) is a sensitive method which enables the detection of small dynamic changes in mRNA levels and gene expression between different samples, for example to examine the effects of an experimental treatment or compare expression in tissue samples from two patient groups [1]. Many advances in the technological platforms in this field have arisen over recent years allowing for high-throughput analysis to scale up and compare greater numbers of samples or analyse multiple targets at the same time, as well as more accurate detection and quantification methods and software [2, 3]. Despite these advances, small variations in technical procedures can result in misinterpretation of qPCR results. Sources of variation include but are not limited to; quantity of starting material, efficiency of reverse transcriptase, cDNA sample loading and variation arising from the quality and integrity of RNA in the sample following extraction and purification procedures [4]. The MIQE (minimum information for publication of quantitative real-time PCR experiments) guidelines define the most important steps from RNA to qPCR to minimize errors and the minimum set of information required to evaluate the reliability of qPCR data [5].

Due to these sources of error, relative expression cannot simply be based on the amount of starting material. To correct for non-specific experimental variation, qPCR results need to be normalized against one or more internal control or reference genes (RGs), formerly referred to as ‘housekeeping genes’ since they are typically genes that are constitutively expressed and required for the maintenance of basic cellular functions [6]. MIQE guidelines highly recommend using at least two different RGs to normalize the expression of the gene under investigation [5]. Using the geometric mean of two or more RGs, has been shown to result in more accurate comparisons and quantification between sample groups [4, 7]. However, the accountability and reproducibility of relative expression analyses depends greatly on whether the expression of the RGs themselves, also remains stable. It has been shown that the expression of a number of traditional RGs varies depending on species, tissue type, cell line, developmental stage and/or in response to experimental treatments [8, 9]. Hence, the utility of some of these classical RGs is limited [10, 11].Therefore, for accurate normalization of gene expression data, it is also important to evaluate the stability of candidate RGs [12].

During pregnancy, the uterus undergoes hyperplasia and significant hypertrophy to accommodate the growing fetus. This growth is largely due to stretch-induced hypertrophy of the myometrial smooth muscle cells [13]. Due to this significant growth, as well as influences from changes in hormone levels and pressure on the uterus, it could be postulated that the expression of some classical RGs such as cytoskeletal proteins or proteins involved in the cell cycle or cell metabolism may also change with gestational age. For example, steroidal hormones have shown to influence expression of some RGs in mouse uterus [14, 15]. Due to the increased stretch on the myometrium, the rate of uterine growth is also potentially changed in the case of twin pregnancies. When the research question being addressed is comparing gene expression in different pregnancy groups, such as comparing different gestational time points e.g. term (≥ 37 weeks) and preterm (< 37 weeks) gestations or pregnancy groups e.g. singleton and twin pregnancies, it is important that the RGs selected for normalisation do not change with advancing gestation.

There have been few studies which report a systematic screening of RGs to examine their stability in human myometrium [16, 17], but none in pregnancy. Studies to date in pregnant human reproductive tissues have largely focussed on the placenta [18, 19]. Where myometrial tissue has been investigated, studies have either used non-pregnant tissues and/or have involved culturing of cells [16, 17]. Hence, these experimental conditions are quite different to fresh, pregnant myometrium and one cannot simply extrapolate.

This study undertook a systematic quantitative review of the gene expression literature to identify the most commonly used RGs in studies of pregnant human myometrium. The stability of their expression was validated in fresh samples of myometrium from both singleton and twin pregnancies, as well as from across a range of gestations, before determining the most suitable RGs for qPCR in pregnant human myometrium. For this, a panel of 12 RGs and two common normalisation algorithms, geNorm [4] and NormFinder [20] was used, which employ different approaches to evaluate the suitability a gene or gene set as a normalisation factor.

Using geNorm, the optimal number of reference genes required for accurate normalisation using a pairwise variation (V_(n/n+1)_) approach was also determined. Finally, the relative expression of the gene encoding the oxytocin receptor (*OXTR*), which is known to be highly expressed in pregnant myometrium, predominantly after 37 weeks gestation [21, 22], was examined to highlight the impact of RG selection and validate the outcomes from the geNorm and Normfinder tests.

## Methods

### Identifying previously reported reference genes

In July 2020, using the NCBI library and PubMed, the term, ‘*human myometrium gene expression’* was searched. The search was restricted to original article publications within the last 14 years (2006–2020) and those involving pregnant and non-pregnant *human* myometrium but excluded studies of myometrial pathology e.g. leiomyomas, uterine fibroids and myometrial invasion e.g. in endometrial cancers. Studies of fresh myometrial tissue, primary myometrial cells and explants and immortalised myometrial cell lines e.g. PHM1-41 and hTERT-HM were included.

The identified studies were then manually examined to confirm that the study involved *human* myometrium and used quantitative PCR (qPCR) techniques. The RG or genes used for normalisation purposes were identified and any evidence of RG validation was noted.

### Human tissue collection

Biopsies of human myometrium (n = 14) were collected during pre-labour elective Caesarean Section (CS) delivery at Liverpool Women’s Hospital NHS Foundation Trust. The project was approved by the Local Research Ethics Committee (REC Ref. 10/H1002/49+5) and by the Trust Research and Development manager and University Institutional review board. All women gave written informed consent prior to their operation. Delivery by CS was for the following indications: maternal request, breech presentation, previous CS delivery or placenta previa. Delivery was between 34 and 40^+5^ week’s gestation with singleton (n = 7) or twin (n = 7) pregnancy. Term delivery was considered ≥ 37 weeks gestation and delivery before 37 weeks was considered preterm.

Myometrium was obtained from the upper lip of the lower uterine incision site following delivery of baby/ies prior to oxytocin administration and placed in cooled Hanks balanced salt solution. After transporting to the adjacent laboratory, samples were micro dissected and cleaned of decidua, fetal membrane and scar tissue (if present) under stereomicroscope [23]. The cleaned myometrium was then immediately placed into RNA Later for 24 h at 4 °C before being frozen in liquid nitrogen and stored at – 80 °C until subsequent RNA extractions and qPCR.

### RNA isolation and cDNA synthesis

Total RNA was isolated from 80-90 mg of thawed tissue by homogenisation in TRIzol Reagent (Life Technologies) using an IKA Ultra Turrax homogenizer (Cole-Palmer) and extracted using the Trizol Plus RNA Purification Kit (Life Technologies). RNA samples were purified using the TURBO DNA-*free* kit (Thermo Fisher Scientific, UK) to remove any contaminating genomic DNA. RNA concentration and quality were assessed by QuBIT (Invitrogen) and NanoDrop ND-1000 spectrophotometer (ThermoFisher Scientific, UK) respectively. A_260/_A_280_ absorbance ratios were > 1.8 and A_260_/A_230_ ratios were between 2.0 and 2.2.

Complementary DNA (cDNA) was synthesised from 500 ng total RNA in 20 µL reaction volumes using the AMV First Strand cDNA synthesis kit (New England Biolabs, UK) with random primer mix. The reverse transcription reaction was carried out in a Bio-Rad T100 Thermal Cycler (Bio-Rad Laboratories, Inc.) with the following conditions: 25 °C for 5 min, 42 °C for 60 min and 80 °C for 5min. The final product was diluted to correspond to 10 ng initial RNA input/µL and was stored at − 20 °C.

### Selection of candidate reference genes

A total of 12 candidate RGs based on those used in previous studies of human myometrium (including from fresh tissue, primary cells and immortalised cells) and emerging RGs in other tissues types were used to identify the most suitable RGs for gene expression analysis using qPCR. The candidate RGs were β-actin (*ACTB*), glyceraldehyde-3-phosphate dehydrogenase (*GAPDH*), ubiquitin C (*UBC*), β2-microglobulin (*B2M*), tyrosine 3-monooxygenase (*YWHAZ*), ribosomal protein L13A (*RPL13A*), 18S rRNA (*18s*), cytochrome c-1 (*CYC1*), eukaryotic translation initiation factor 4A, isoform 2 (*EIF4A2*), succinate dehydrogenase complex (*SDHA*), topoisomerase (DNA) I (*TOP1*) and ATP synthase, (*ATP5B*). The genes selected also covered a wide variety of cellular functions to minimise the risk of co-regulation between genes.

### Quantitative real-time PCR (qPCR)

Quantitative PCR was performed in 96-well plates on a Bio-Rad CFX connect instrument (Bio-Rad Laboratories, Inc). using Precision® FAST qPCR Mastermix (Primer design, UK) and SYBR green chemistry. Each qPCR reaction contained 10 µl of 2X Precision® FAST Mastermix, 10 ng of cDNA, 0.3 µM of each primer and PCR-grade water up to total volume of 20 µl. Thermal cycling followed manufacturers recommendations and composed of an initial enzyme activation step at 95 °C for 2 min followed by 40 cycles of denaturation at 95 °C for 5 s and annealing and extension at 60 °C for 20 s during which data was collected. Afterwards, the dissociation (melt) curve was obtained by melting the amplicon from 60 to 95 °C and was used to assess the specificity of the primers and ensure that amplification of non-specific products did not occur. All reactions were performed in triplicate together with a negative control (no template control, NTC). A sample maximization strategy was used in which the number of samples per plate was maximized rather than the number of genes to reduce further technical variation [24].

All primers were purchased from Primer Design, UK. Accession numbers,anchor nucleotides and amplicon length for the assays are presented in Supplementary Table 1. Primer sequences remain PrimerDesign proprietary information and are not available. The quality of the primer pairs was examined using a 5-fold dilution series of human myometrial cDNA and standard curves analyzed and the amplification efficiency determined using Bio-Rad CFX Manager software (Bio-Rad Laboratories, Inc). The mean threshold cycle for each gene was calculated from triplicate reactions, and then corrected for the efficiency of the reaction. Amplification efficiencies > 90% and correlation co-efficient ≥ 0.99 were considered acceptable. All NTC samples had Cq values at least 5 cycles higher than the highest Cq of the unknowns.

### Analysis of gene stability and variability using geNorm and NormFinder

#### GeNorm analysis

The geNorm algorithm module within qbase^+^ software (version 3.0, Biogazelle, Ghent, Belgium) was used to analyse the stability of the candidate RGs and to determine the minimum number of recommended RGs required for optimal normalisation. Quantification cycle (Cq) data for each candidate RG and for each sample were imported and Cq data was examined to ensure all values were < 30 and that replicate variability Cq standard deviation was < 0.1. GeNorm analysis was performed on the arithmetic mean of the replicate values. The algorithm evaluates RG suitability based on two quality parameters: the stability value (M) and the coefficient of variation (CV) of the normalized RG expression levels [4, 24]. The gene expression stability measure (M) for an RG, is calculated as the average pairwise variation for that gene, against all other tested RGs. Stepwise elimination of genes with the highest M values allows ranking of the tested genes according their expression stability. Genes with the lowest M value (M < 0.5) are considered the most stably expressed across the dataset. Genes with CV < 0.2 fail quality control and are also not considered suitable.

GeNorm also determines the optimal number of RGs required for normalization in comparative gene expression analyses using V parameter and is based on pairwise variation analysis (*V*_*n/n+1*_) between sequential RGs, starting with those genes with the lowest M values, and stepwise inclusion of the next most stable remaining RG. Generally, V < 0.15 is considered the threshold value below which an additional RG is not required for accurate normalization [4].

#### NormFinder analysis

For NormFinder analysis [20] the Microsoft Excel-based application, freely available from http://moma.dk/normfinder-software was used. Cq values were converted into relative quantities via the ∆Ct method using the sample with the lowest Cq as a calibrator and were imported into the application. Similarly to geNorm, NormFinder estimates the variation in expression between the candidate RGs, based on sample subgroups (singleton and twin myometrium in this study) and the estimation of intra- and inter-group variation in expression levels. NormFinder then ranks the genes according to their stability (S). Values closest to 0 indicate the best genes or the most stable genes to be used as RGs. NormFinder also calculates the stability value for the best combination of genes [20].

### Evaluation of reference genes and statistical analysis

To validate the selected RGs for normalization, the relative expression of the oxytocin receptor gene, *OXTR,* was compared after normalising to the most and least stable RGs and was calculated by the ∆Ct Method, derived as a modification of the 2^−∆∆Ct^ approach [25]. The applied ∆Ct method uses the difference between reference and target Ct values for each sample, (Ratio (reference/target) = 2 ^Ct(reference)−Ct(target)^).

Data are presented as mean + 95% CIs. Statistical analyses were performed by unpaired Student’s *t* test between singleton and twin and preterm and term groups, using GraphPad Prism 5.0. Results were considered statistically significant when *P* < 0.05.

## Results

### Identification of the most commonly used reference genes in human myometrial research

Searching the term ‘*human myometrium gene expression*’ yielded 997 results (see Suppl. Fig. 1). Limiting to original journal articles published in Pubmed between 2006 and 2020 and ‘human’ resulted in 580 articles. Exclusion of studies of myometrial pathology and non-human studies, as inferred from the article title, and review articles returned 189 items.

Manual inspection of these articles also resulted in the exclusion of 49 articles due to studies being in rodents which was not inferred from the title (n = 3), studies using other expression techniques and not qPCR (n = 25 including; ELISA, western blot, ChIP), semi-quantitative RT-PCR (n = 9); RNA Seq (n = 3); meta-analysis of microarray and/or RNA Seq data (n = 2) and no RGs stated and which could not be inferred from the figures or list of primers used (n = 5). The final 140 articles were identified, full text accessed, and the RG(s) used noted. The full table of papers identified, and data extracted are provided as supplementary information (Supplementary Table 2 ).

The search strategy identified 25 different RGs or alternative methods being used for normalisation across the 140 publications (Table 1). *GAPDH, 18s*, *ACTB* and *B2M* were the most commonly reported RGs, with one or more being used in over 70% of the studies examined.

**Table 1:** Different genes and their frequency of use as reference genes in published human myometrial gene expression studies

Gene symbol	Name	No. of studies	Percentage
*GAPDH*	Glyceraldehyde-3-phosphate dehydrogenase	57	31.49
*ACTB*	β-Actin	29	16.02
*18s rRNA*	18S ribosomal RNA	28	15.47
*B2M*	Β2-Microglobulin	13	7.18
*SHDA*	Succinate dehydrogenase, subunit A	9	4.97
*RPLP0*	Ribosomal protein, large P0	7	3.87
*YWHAZ*	Tyrosine 3-Monooxygenase	6	3.31
*PPIA*^*#*^	Peptidylprolyl Isomerase A	4	2.21
*Other*	Alien primer, cDNA	3	1.66
*RNU6*	U6 Small Nuclear 1	3	1.66
*GUSB*^*#*^	β-glucuronidase	3	1.66
*POLR2A*	RNA polymerase II	3	1.66
*EEF1A1*	Elongation factor 1-alpha 1	2	1.10
*RPL19*	Ribosomal Protein L19	2	1.10
*RPL30*	Ribosomal Protein L30	2	1.10
*5S rRNA*	Ribosomal 5S RNA	1	0.55
*ACTA1*	α-Actin	1	0.55
*CALD1*^*#*^	H-caldesmon	1	0.55
*TOP1*	DNA topoisomerase	1	0.55
*PSMD2*	26S proteasome non-ATPase regulatory subunit 2	1	0.55
*RPL27*	Ribosomal Protein L27	1	0.55
*LRP10*	Low-density lipoprotein receptor-related protein 10	1	0.55
*ARHGDIA*^*#*^	Rho GDP Dissociation Inhibitor Alpha	1	0.55
*RPL13A*	Ribosomal Protein L13a	1	0.55
*RPL32*	Ribosomal Protein L32	1	0.55

^#^Denotes that an alternative pseudonym may have been used in the original article

Of the 140 studies identified, only 34 (24.2%) employed more than one RGs and only 18 (12.8%) stated that they had validated their choice of RG, either by confirming their stability under the different experimental conditions or by selecting from a panel of candidate RGs (see Suppl. Table 2). Three studies employed alternative strategies for normalisation involving either an exogenous (or alien) reference RNA molecule that is nonhomologous to any known (human) nucleic acids, which is spiked into the RNA sample and all mRNA abundance data are then expressed relative to the alien reference RNA, or examined absolute qualification using the standard curve method and not the comparative Ct method, and cDNA as a calibrator. Only two studies included twin myometrium, but the choice of RG (*GAPDH* in both studies) was not validated.

**Table 2:** Gene stability values as determined by NormFinder and geNorm algorithms (rankings in parenthesis)

Gene name	NormFinder (S-value)	geNorm (*M* value)
***CYC1***	**0.018 (1)**	**0.324 (1)**
***ATP5B***	**0.024 (2)**	**0.334 (3)**
***YWHAZ***	**0.028 (3)**	**0.329 (2)**
*GAPDH*	0.056 (4)	0.383 (4)
*EIFA2*	0.062 (5)	0.403 (5)
*SDHA*	0.064 (6)	0.404 (6)
*RP13A*	0.068 (7)	0.406 (7)
*TOP1*	0.076 (8)	0.418 (8)
*ACTB*	0.094 (9)	0.452 (9)
*UBC*	0.105 (10)	0.497 (10)
*B2M*	0.144 (11)	0.642 (11)
*18S*	0.151 (12)	0.682 (12)

Values in bold represent the most stable genes

### Expression stability according to geNorm

Twelve different RGs were selected based on those reported most frequently in the literature (*GAPDH, ACTB, 18s and B2M*), those used moderately (*YWHAZ and SDHA*), infrequently *(TOP1 and RPL13A*) and four genes (*EIF4A2, CYC1, ATP5B and UBC*) which were not identified in the literature search but have been used elsewhere in non-pregnant myometrium or in other tissues.

**Fig. 1:**
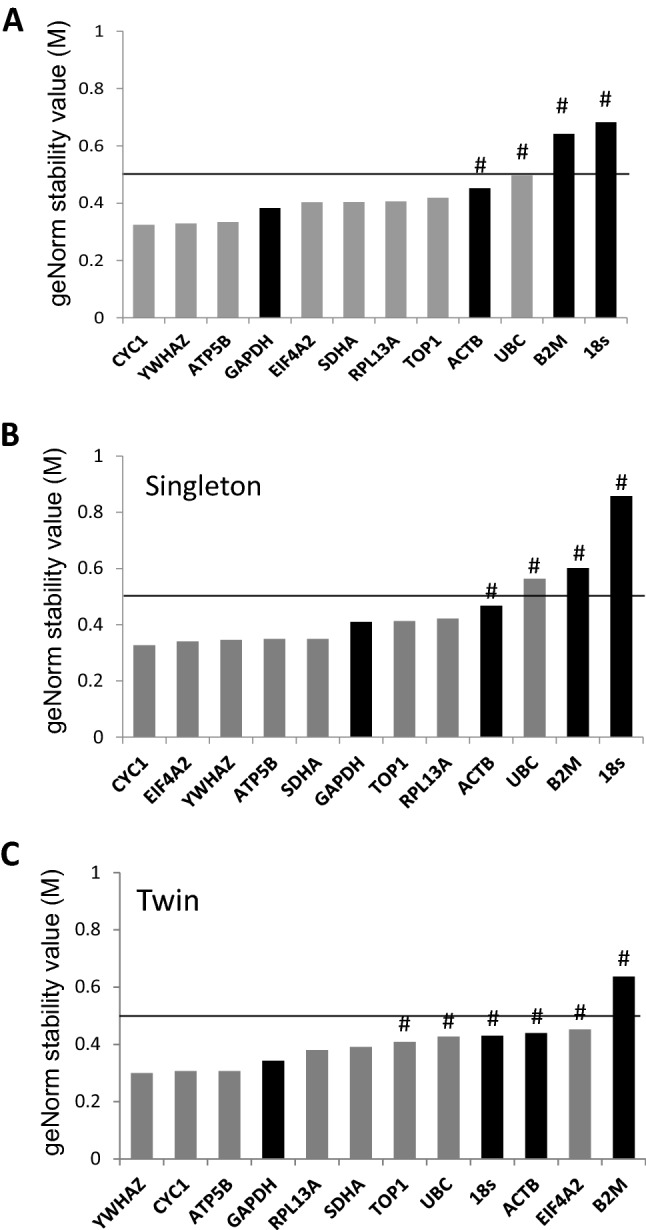
GeNorm analysis of the candidate reference genes. GeNorm stability values (M) of the 12 candidate references genes from all samples of pregnant human myometrium (**a**), samples from singleton pregnancies (**b**) and samples from twin pregnancies (**c**). More stably expressed genes are positioned on the left, less stable on the right. The four classical reference genes are highlighted in black. M < 0.5 was used as stability threshold, ^#^indicates unsuitability due to CV value > 0.2

According to the geNorm algorithm, only *GAPDH* from the frequently used RG group (*ACTB, GAPDH, 18S and B2M*) had a suitable M value and CV value which was below the recommended threshold values of instability (M < 0.5, CV < 0.2), unlike *18s* and *B2M* whose M values were > 0.5 and *ACTB* whose CV value was > 0.2. (Fig. 1a). This result indicates that three of the four classical RGs (*ACTB, 18s and B2M*) do not meet the criteria to be used as RGs in pregnant human myometrium gene expression assays. The geNorm algorithm ranked *CYC1, ATB5B and YWHAZ* as the most stable and hence their use in human myometrial gene expression studies would be recommended.

The samples were then stratified according to pregnancy group i.e. twin or singleton and geNorm analysis was performed to calculate M values for each candidate RG within each group independently. *CYC1, EIF4A2* and *YWHAZ* were found to be the most stable RGs in singletons, whilst *18s, B2M* and *ACTB* as well as *UBC* were again found to be least stable and not suitable due to M > 0.5 or CV > 0.2 (Fig 1b). For twin myometrium, the most stable RGs were *YWHAZ, CYC1* and *ATP5B.* Only *B2M* failed the M stability threshold, however, many others (*EIFA2, ACTB, 18s, UBC* and *TOP1*) were not suitable due to high CV values (Fig. 1c).

### Expression stability according to NormFinder

The NormFinder algorithm detected *CYC1* and *ATB5P* as the genes with the highest stability values (S = 0.018 and 0.024 respectively) suggesting they are the best combination of genes to normalise expression data. The application also found *18s* and *B2M* as the least stable RGs (Table 2).

When comparing the stability rankings of the 12 candidate genes between the two algorithms, there was strong agreement between the two approaches (Table 2). The rankings were identical apart from *ATP5B* and *YWHAZ* which were in positions 2 and 3 in NormFinder and reversed in geNorm.

### Pairwise variation to determine optimal number of reference genes

**Fig. 2:**
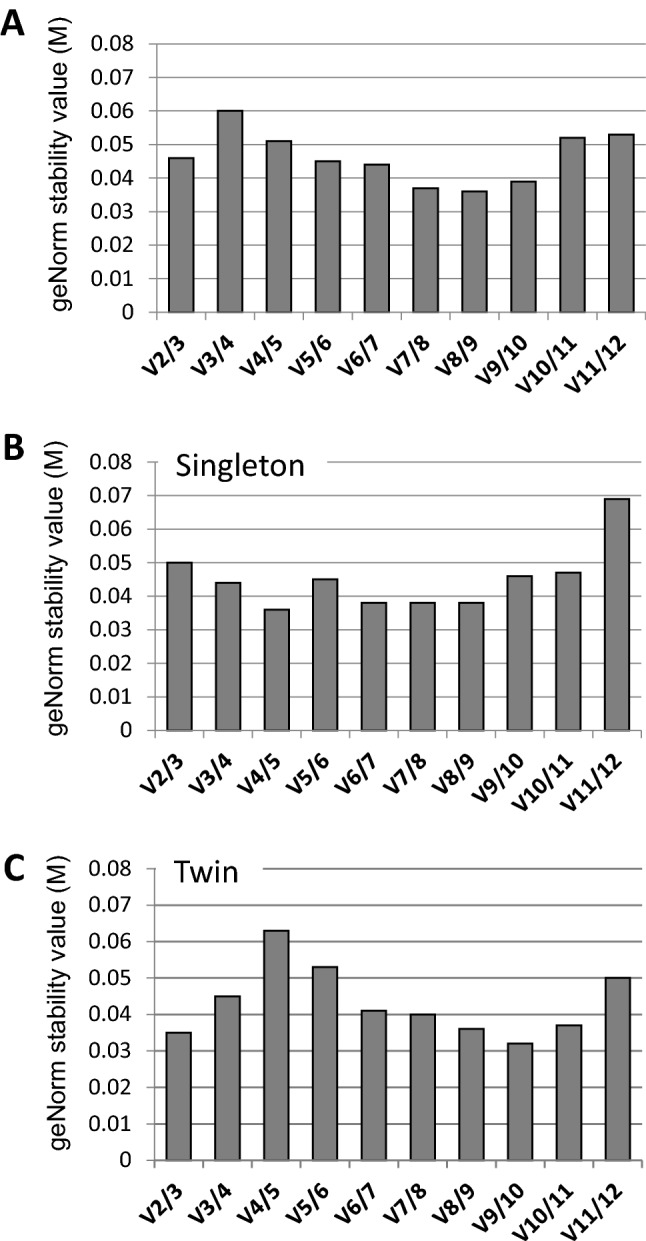
Pairwise variation analysis of the candidate reference genes. The optimal number of reference genes required for reliable normalisation was determined by pairwise variation (V) for all samples combined (**a**) or according to pregnancy group; singleton (**b**) and twin (**c**). Each bar represents the change in normalisation accuracy when adding more reference genes stepwise, according to their stability in Fig 2a. e.g. V2/3 represents the pairwise variation in a 2 vs. 3 reference genes comparison, V3/4 represents pairwise variation in a 3 vs. 4 gene comparison. Pairwise variation value threshold was set at 0.15. Data indicate two reference genes for normalisation is sufficient for all groups

After identification of the most stable genes, the optimal number of RGs for accurate normalisation was determined using the pairwise variation function in geNorm. Pairwise variation values (*V*) were calculated and the recommended threshold value of *V* < 0.15 was used, below which further additional RGs would not be required for accurate normalisation. As shown in Fig. 2a, V_2/3_ was less than 0.15 (V_2/3_ = 0.046) for singleton and twin samples combined and two reference genes were sufficient for correct normalisation. When examined independently Fig. 2b and c, pairwise variation for singleton and twin groups showed that the optimal number of reference targets is also two (V_2/3_ = 0.050 and V_2/3_ = 0.035 respectively). There was only a modest decrease in *V* values with the addition of more RGs and hence, there is no further benefit in adding more RGs to the normalisation process.

Across all analyses and both normalisation tools, *CYC1*, *YWHAZ and ATB5P* were consistently the most stable RGs. The optimal normalisation factor in human myometrium can therefore be calculated as the geometric mean of two of these RGs.

### Evaluation of *CYC1* and *YWHAZ* as reference genes

In order to evaluate the results and determine the effect of normalising target gene expression to different RG sets, the relative expression of *OXTR* in the human myometrial samples using two different combinations of RGs was compared. For each sample, qPCR data were normalised to the geometric mean to two of the top consistently stable genes, *CYC1* and *YWHAZ* and to the geometric mean of *18s* and *B2M*, the least stable genes according to both geNorm and NormFinder. These genes are also two of the most extensively used RGs in the myometrial literature. The relative expression of *OXTR* between pregnancy groups i.e. between singleton and twin pregnancies and between gestation groups i.e. preterm and term groups was compared.

**Fig. 3:**
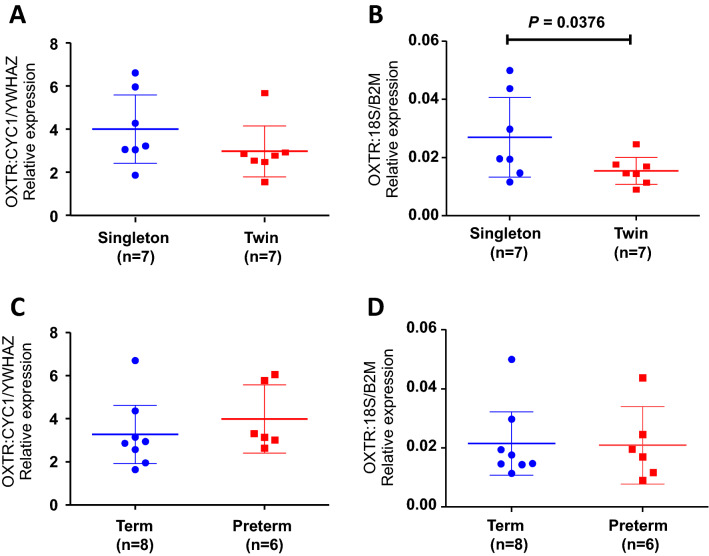
The effect of choice of reference gene set on the relative expression of *OXTR* in pregnant human myometrium. *OXTR* expression was normalised to either the geometric mean of the most stable (*CYC1* and *YWHAZ*) or the least stable (*18s* and *B2M*) reference genes as determined by geNorm and NormFinder. Relative levels of *OXTR* were determined and compared between singleton and twin myometrium (**a** and **b**) and term and preterm samples (**c** and **d**). Data indicate Mean + 95% Cis and were compared by unpaired Student’s t-test. The mean gestational age for singleton samples is 263.8 (± 5.5) days and 259.3 (± 3.2) days for twins (*P* = 0.48). The mean gestational age for term samples was 269.1 (± 2.8) days and 251.5 (± 3.0) days for preterm samples (*P* < 0.001)

When using *CYC1* and *YWHAZ* as RGs, there was no difference in *OXTR* expression between singleton and twin myometrium (Fig. 3a) whereas normalising to *18s* and *B2M* showed that *OXTR* expression was significantly higher in singleton myometrium compared to twins (Fig. 3b). The expression of *OXTR* however, did not differ significantly between preterm and term groups, regardless of which RGs were used to normalise expression (Fig. 3c and d).

## Discussion

qPCR can be an accurate and robust method to study gene expression profiles across different experimental settings and between different sample groups. The reliability of results however is strongly dependent on the accurate normalization procedure which includes the selection of the most appropriate reference genes in terms of their stability and number [4].

When examining changes in gene expression in tissues, the pregnant myometrium presents a unique set of challenges, largely based around changes which occur during gestation, and that the expression of genes, including RGs, may change as pregnancy progresses, as has been observed as the myometrium transitions into labour e.g. [26–28]. This could be due to changes in steroid hormone levels, growth hormones or physical changes resulting from the growing fetus(es) which adds stretch or pressure to the uterus, as described in more detail elsewhere [13, 29, 30]. To accurately determine changes in target gene expression, it is critical then that the expression level of RGs, which act as a normalising factor, also remain stable. Using human myometrial samples from singleton and twin pregnancies and from a range of gestations (preterm and term), this study evaluated the stability of the most commonly used RGs in human myometrial qPCR studies, as well as used two commonly used normalisation algorithms, geNorm and NormFinder, to identify other (more) suitable candidate RGs.

In searching the literature for studies of gene expression in human myometrial tissues, cells and explants, *GAPDH, 18s, B2M* and *ACTB* were found to be the most commonly used RGs. Despite this, expression stability analysis using two normalisation tools, revealed that, *18s*, *B2M* and *ACTB* showed poor stability in the tissues. However, although not the most stable of genes tested, *GAPDH* was found to be a suitably stable RG for human myometrial expression studies. This is somewhat reassuring, since *GAPDH* was also the RG shown to be most widely used in the literature (Table 1 and Suppl Table 2). The most stable RGs were *CYC1*, *YWHAZ and ATB5P*.

Pairwise variation showed that two RGs are adequate for accurate normalisation. That the stability rankings of the candidate genes derived from both NormFinder and geNorm analyses are in high agreement, strongly supports using the geometric mean of *CYC1* and *YWHAZ or ATP5B* for normalising gene expression in fresh human myometrium samples. Whilst *CYC1, YWHAZ* and *ATP5B* were found to be most stable, other genes (i.e. *TOP1, RPL13A, SDHA, EIF4a2*, and *GAPDH*), were also stable. Normalising expression to the geometric mean of a combination of two of these genes would also be sufficient.

These results somewhat agree with a large RG analysis study by Almeida et al. [16]. They examined the stability of 51 different genes in (non-pregnant) human myometrium and matched leiomyoma samples and during different phases of the menstrual cycle. In their findings, they also excluded traditional RGs such as *18s*, *ACTB* and *B2M* since they did not meet the target threshold stability of 0.5 and 0.2 for M and CV respectively. However, in contrast to this study, they also had to exclude *GAPDH* for this reason. Both *18s* and *B2M* have also been shown as being less stable in myometrium from cyclical and pregnant cows during the oestrus cycle and gestation [31], hence their suitability as RGs in myometrium is questionable across species.

The results from this study however, differ to those from Areanas-Hernandez and Vega Sanchez (2013) [17]. They found *18s* to be a suitable RGs for normalisation in human myometrium. However, they were comparing myometrium obtained from patients undergoing hysterectomies due to uterine leiomyomas and therefore the myometrium was also likely to be non-pregnant. Furthermore, the experimental approach here also differed in that the analyses were done using fresh tissues, whilst they were using cultured primary cells and comparing the effects of mitogen stimulation on RG stability.

This study also demonstrates that target gene expression levels can vary according to choice of RGs. *OXTR* expression was shown to be is significantly different between singleton and twin myometrium depending to the RG set used. In a recent study using RNA Sequencing [32] which aimed to identify differences in gene expression between singleton and twin myometrium, no difference in the expression of OXTR was found. Hence, one would predict that the data normalised to *CYC1* and *YWHAZ* rather than *18s* and *B2M* in this study is reliable. The difference in findings emphasizes the importance of choosing the correct set of RGs in gene expression experiments. Further, the range of relative *OXTR* expression within each group was also large, which was true for both RG sets used. This again strengthens the need for using appropriate RGs in gene expression studies.

This study focussed on fresh tissues and did not examine primary cells or immortalised cells in culture. It is well known that culturing of smooth muscle cells changes their phenotype towards a more synthetic and secretory one [33, 34], hence the stability of RG may also change under culture conditions. This study also did not examine labouring samples which is a limitation. Therefore, further analysis should be performed to validate these RGs in these different model systems. The sample maximisation strategy chosen enabled all samples to be examined simultaneously and reduce technical variation, however, this limited the sample size. To improve the study’s robustness, a range of gestations as well as singleton and twin myometrium was used, increasing the variability between samples. The RG set identified should therefore be applicable to many sample populations.

It was encouraging to find that some more recent studies of human myometrium have adopted the use of more than one RG or have taken steps to validate their choice of RG prior to qPCR analysis (see Suppl Table 2). This is likely as a result of the MIQE guidelines being more widely disseminated and adopted [12]. None of the studies identified however, have used *CYC1* as a normalising factor, hence this gene presents a novel alternative RG for use in human myometrial studies. Others have applied other methods for normalisation such as alien spike-ins (see Suppl Table 2). Adding a spike-in or alien RNA to samples prior to reverse transcription, is an approach which is typically used to enable quality control of the RNA isolation, cDNA synthesis and PCR amplification steps (depending on where in the process it is added) [35–37]. However, so-called spike-ins can also be used as a pseudo-reference gene with which the expression of a target gene can be normalised to the external RNA [36]. However, its accuracy also depends on the precise addition of spike-in RNA to each sample [38]. Currently, normalisation to multiple RGs is considered the gold standard approach by the MIQE guidelines, which also insist that that validation of RGs is performed [5].

In conclusion, this study identified a set of RGs, suitable for comparing fresh human myometrial samples which encompass a range of gestational ages (from 34 to 40 weeks gestation) and multiplicity of pregnancy (singleton and twin). Across all samples and both normalisation tools, *CYC1*, *YWHAZ* and *ATP5B* were found the be the most stable genes. The minimum number of RGs to be included in the normalisation of qPCR data across all analyses was found to be two. Hence, using the geometric mean of *CYC1* and *YWHAZ or ATP5B* when normalising gene expression data in pregnant human myometrium is advised. Genes such as *18s, ACTB* and *B2M* which are commonly employed as RGs, were found to be ranked amongst the most variable candidate genes and therefore their use in human myometrial research is not recommended.

## Electronic supplementary material

Below is the link to the electronic supplementary material.

Suppl. Fig. 1 Study eligibility flow chart. The number (n) of studies included and excluded at various stages of the quantitative review process (eps 3647 kb)

(DOCX 14 kb)

(DOCX 39 kb)

## Data Availability

Any data not presented can be made available upon request.
