# Supplementary material for: Identification and validation of suitable reference genes for quantitative real-time PCR gene expression analysis in pregnant human myometrium
Source: Mol Biol Rep. 2021 Jan 1;48(1):413–23. doi: 10.1007/s11033-020-06066-2 (PMC7884357; doi:10.1007/s11033-020-06066-2)
Supplement: Supplementary file 2 — (DOCX 14 kb) [file 11033_2020_6066_MOESM2_ESM.docx]

**Suppl. Table 1. List of primers used for the 12 reference candidate genes and OXTR for the qPCR analysis in pregnant human myometrium**

| **Gene name** | **Gene description** | **Accession number** | **Amplicon length** | **Anchor nucleotide** |
| --- | --- | --- | --- | --- |
| *ACTB* | β-actin | NM_001101 | 92 | 1195 |
| *GAPDH* | Glyceraldehyde-3-phosphate dehydrogenase | NM_002046 | 110 | 989 |
| *UBC* | Ubiquitin C | NM_021009 | 137 | 61 |
| *B2M* | β2-microglobulin | NM_004048 | 114 | 332 |
| *YWHAZ* | Tyrosine 3-monooxygenase | NM_003406 | 120 | 2572 |
| *RPL13A* | Ribosomal protein L13A | NM_012423 | 153 | 673 |
| *18s* | 18S ribosomal RNA | M10098 | 93 | 234 |
| *CYC1* | Cytochrome c-1 | NM_001916 | 145 | 910 |
| *EIF4A2* | Eukaryotic translation initiation factor 4A, isoform 2 | NM_001967 | 113 | 895 |
| *SDHA* | Succinate dehydrogenase complex | NM_004168 | 120 | 953 |
| *TOP1* | Topoisomerase (DNA) I | NM_003286 | 141 | 2361 |
| *ATP5B* | ATP synthase | NM_001686 | 119 | 1115 |
| *OXTR* | Oxytocin receptor | NM_000916 | 105 | 3684 |
