# Supplementary material for: Identification and validation of suitable reference genes for quantitative real-time PCR gene expression analysis in pregnant human myometrium
Source: Mol Biol Rep. 2021 Jan 1;48(1):413–23. doi: 10.1007/s11033-020-06066-2 (PMC7884357; doi:10.1007/s11033-020-06066-2)
Supplement: Supplementary file 3 — (DOCX 39 kb) [file 11033_2020_6066_MOESM3_ESM.docx]

**Suppl. Table 2 Studies identified from the literature search of ‘*human myometrium gene expression’***

| **Study title** | **Pubmed article ID #** | **Reference gene 1** | **Reference gene 2** | **Reference gene 3** | **Evidence of RG validation** |
| --- | --- | --- | --- | --- | --- |
| Progesterone receptor-A and -B have opposite effects on proinflammatory gene expression in human myometrial cells: implications for progesterone actions in human pregnancy and parturition. | 22419721 | ACTA |  |  |  |
| The contribution of Kv7 channels to pregnant mouse and human myometrial contractility. | 20132415 | ACTB | GAPDH | B2M | YES |
| Responsivity to PGE2 labor induction involves concomitant differential prostaglandin E receptor gene expression in cervix and myometrium. | 26400315 | ACTB | GAPDH |  | YES |
| Inhibition of Inflammatory Changes in Human Myometrial Cells by Cell Penetrating Peptide and Small Molecule Inhibitors of NFκB. | 30619324 | ACTB | GAPDH | B2M |  |
| Endogenous hydrogen sulfide contributes to uterine quiescence during pregnancy. | 28188160 | ACTB | GAPDH |  |  |
| Expression of cystathionine β-synthase and cystathionine γ-lyase in human pregnant myometrium and their roles in the control of uterine contractility. | 21886822 | ACTB | GAPDH |  |  |
| Pro-labour myometrial gene expression: are preterm labour and term labour the same? | 18367515 | ACTB |  |  | YES |
| The Transcription Factor Interferon Regulatory Factor-1 (IRF1) Plays a Key Role in the Terminal Effector Pathways of Human Preterm Labor. | 26674566 | ACTB |  |  | YES |
| Bitter taste receptors as targets for tocolytics in preterm labor therapy. | 28559440 | ACTB |  |  |  |
| Decreased DNA Methylations at the Progesterone Receptor Promoter A Induce Functional Progesterone Withdrawal in Human Parturition. | 24401475 | ACTB |  |  |  |
| Differential expression of the metalloproteinase MMP3 and the alpha5 integrin subunit in human myometrium at labour. | 17664256 | ACTB |  |  |  |
| Expression of Matrix Metalloproteinases in the Mouse Uterus and Human Myometrium During Pregnancy, Labor, and Preterm Labor. | 28950743 | ACTB |  |  |  |
| Expression of prothrombin and protease activated receptors in human myometrium during pregnancy and labor. | 17901076 | ACTB |  |  |  |
| Ghrelin in the human myometrium. | 20509935 | ACTB |  |  |  |
| Human cathelicidin antimicrobial protein 18 (hCAP18/LL-37) is increased in foetal membranes and myometrium after spontaneous labour and delivery. | 25435436 | ACTB |  |  |  |
| Misoprostol modulates the gene expression prostaglandin E2 and oxidative stress markers in myometrial cells. | 27647508 | ACTB |  |  |  |
| Myometrial oxytocin receptor mRNA concentrations at preterm and term delivery - the influence of external oxytocin. | 19347709 | ACTB |  |  |  |
| Peroxisome proliferator-activated receptor alpha expression changes in human pregnant myometrium. | 23144166 | ACTB |  |  |  |
| Progesterone signaling in human myometrium through two novel membrane G protein-coupled receptors: potential role in functional progesterone withdrawal at term. | 16484338 | ACTB |  |  |  |
| Regional expression of prostaglandin E2 and F2alpha receptors in human myometrium, amnion, and choriodecidua with advancing gestation and labor. | 16707767 | ACTB |  |  |  |
| The onset of labor alters corticotropin-releasing hormone type 1 receptor variant expression in human myometrium: putative role of interleukin-1beta. | 17431005 | ACTB |  |  |  |
| Upregulation of PSCDBP, TLR2, TWIST1, FLJ35382, EDNRB, and RGS12 gene expression in human myometrium at labor. | 18497345 | ACTB |  |  |  |
| Differential expression of AP-1 proteins in human myometrium after spontaneous term labour onset. | 24784710 | ACTB |  |  |  |
| Modulation of human uterine smooth muscle cell collagen contractility by thrombin, Y-27632, TNF alpha and indomethacin. | 19133144 | ACTB |  |  |  |
| Integrated microRNA and mRNA network analysis of the human myometrial transcriptome in the transition from quiescence to labor. | 29447339 | B2M | RPL30 |  |  |
| Inhibition of PIM1 kinase attenuates inflammation-induced pro-labour mediators in human foetal membranes in vitro. | 28333279 | B2M | SDHA |  | YES |
| RAF1 is increased in labouring myometrium and modulates inflammation-induced pro-labour mediators. | 26811545 | B2M | SDHA |  | YES |
| Copper metabolism domain-containing 1 represses the mediators involved in the terminal effector pathways of human labour and delivery. | 26733542 | B2M |  |  | YES |
| Development and validation of primary human myometrial cell culture models to study pregnancy and labour. | 23445904 | B2M |  |  |  |
| Labour is associated with decreased expression of the PGF2alpha receptor (PTGFR) and a novel PTGFR splice variant in human myometrium but not decidua. | 20619365 | B2M |  |  |  |
| Prostaglandin E2 represses interleukin 1 beta-induced inflammatory mediator output from pregnant human myometrial cells through the EP2 and EP4 receptors. | 22517618 | B2M |  |  |  |
| Estrogen receptor (ER) expression and function in the pregnant human myometrium: estradiol via ERα activates ERK1/2 signaling in term myometrium. | 22068927 | CALD1 |  |  | YES |
| Effect of cigarette smoking on mRNA and protein levels of oxytocin receptor and on contractile sensitivity of uterine myometrium to oxytocin in pregnant women. | 24792665 | EEF1A1 |  |  |  |
| Cigarette smoke extract enhances oxytocin-induced rhythmic contractions of rat and human preterm myometrium. | 16885542 | EEF1A1 |  |  |  |
| The study of progesterone action in human myometrial explants. | 27235325 | GAPDH | 18S RNA |  |  |
| Nuclear factor of activated T-cell isoform expression and regulation in human myometrium. | 26238508 | GAPDH | ACTB | B2M | YES |
| MicroRNA-200a serves a key role in the decline of progesterone receptor function leading to term and preterm labor. | 22529366 | GAPDH | RPLP0* |  |  |
| KATP channels are up-regulated with increasing age in human myometrium. | 23369859 | GAPDH |  |  | YES |
| A novel role for FOXO3 in human labor: increased expression in laboring myometrium, and regulation of proinflammatory and prolabor mediators in pregnant human myometrial cells. | 23636809 | GAPDH |  |  |  |
| A novel role for GSK3 in the regulation of the processes of human labour. | 25550525 | GAPDH |  |  |  |
| Adaptive reduction of human myometrium contractile activity in response to prolonged uterine stretch during term and twin pregnancy. Role of TREK-1 channel. | 29577872 | GAPDH |  |  |  |
| Caspase-1 activation is increased with human labour in foetal membranes and myometrium and mediates infection-induced interleukin-1β secretion. | 24238269 | GAPDH |  |  |  |
| Comparative analysis of myometrial and vascular smooth muscle cells to determine optimal cells for use in drug discovery. | 31078743 | GAPDH |  |  |  |
| Control of Progesterone Receptor-A Transrepressive Activity in Myometrial Cells: Implications for the Control of Human Parturition. | 28671036 | GAPDH |  |  |  |
| Cyclic AMP Effectors Regulate Myometrial Oxytocin Receptor Expression. | 27673556 | GAPDH |  |  |  |
| Cyclic AMP enhances progesterone action in human myometrial cells. | 24161591 | GAPDH |  |  |  |
| Differential Effects of Oxytocin Receptor Antagonists, Atosiban and Nolasiban, on Oxytocin Receptor-Mediated Signaling in Human Amnion and Myometrium. | 28188254 | GAPDH |  |  |  |
| Effects of combined progesterone and 17β-estradiol treatment on the transcriptome of cultured human myometrial smooth muscle cells. | 26534934 | GAPDH |  |  |  |
| Epigenetics of human myometrium: DNA methylation of genes encoding contraction-associated proteins in term and preterm labor. | 24571989 | GAPDH |  |  |  |
| Finding lost genes in GWAS via integrative-omics analysis reveals novel sub-networks associated with preterm birth. | 27664809 | GAPDH |  |  |  |
| Forkhead box O1 (FOXO1) in pregnant human myometrial cells: a role as a pro-inflammatory mediator in human parturition. | 23778262 | GAPDH |  |  |  |
| HoxA13 Stimulates Myometrial Cells to Secrete IL-1β and Enhance the Expression of Contraction-Associated Proteins. | 2698235 | GAPDH |  |  |  |
| Interactions between inflammatory signals and the progesterone receptor in regulating gene expression in pregnant human uterine myocytes. | 22435466 | GAPDH |  |  |  |
| Maternal and fetal intrauterine tissue crosstalk promotes proinflammatory amplification and uterine transition†. | 30379983 | GAPDH |  |  |  |
| Opioid mediated activity and expression of mu and delta opioid receptors in isolated human term non-labouring myometrium. | 23051674 | GAPDH |  |  |  |
| Progesterone acts via the nuclear glucocorticoid receptor to suppress IL-1β-induced COX-2 expression in human term myometrial cells. | 23209664 | GAPDH |  |  |  |
| Progesterone and cAMP synergize to inhibit responsiveness of myometrial cells to pro-inflammatory/pro-labor stimuli. | 30118888 | GAPDH |  |  |  |
| Progesterone and the Repression of Myometrial Inflammation: The Roles of MKP-1 and the AP-1 System. | 26280733 | GAPDH |  |  |  |
| Slit2 is decreased after spontaneous labour in myometrium and regulates pro-labour mediators. | 25130654 | GAPDH |  |  |  |
| SLIT3 is increased in supracervical human foetal membranes and in labouring myometrium and regulates pro-inflammatory mediators. | 24286238 | GAPDH |  |  |  |
| The androgen receptor mediates antiapoptotic function in myometrial cells. | 25032861 | GAPDH |  |  |  |
| The effect of trichostatin-A and tumor necrosis factor on expression of splice variants of the MaxiK and L-type channels in human myometrium. | 25076912 | GAPDH |  |  |  |
| The influence of maternal body mass index on myometrial oxytocin receptor expression in pregnancy. | 23653389 | GAPDH |  |  |  |
| The TLR2 ligand FSL-1 and the TLR5 ligand Flagellin mediate pro-inflammatory and pro-labour response via MyD88/TRAF6/NF-κB-dependent signalling. | 24635133 | GAPDH |  |  |  |
| Transcriptomic effects of estradiol treatment on cultured human uterine smooth muscle cells. | 24942541 | GAPDH |  |  |  |
| Beta-Estradiol Regulates Voltage-Gated Calcium Channels and Estrogen Receptors in Telocytes from Human Myometrium. | 29747396 | GAPDH | 18S RNA |  |  |
| Binding loci of RelA-containing nuclear factor-kappaB dimers in promoter regions of PHM1-31 myometrial smooth muscle cells. | 26405173 | GAPDH | ACTB |  |  |
| Myometrial tumor necrosis factor-α receptors increase with gestation and labor and modulate gene expression through mitogen-activated kinase and nuclear factor-κB. | 22228740 | GAPDH | ACTB |  |  |
| Characterization of anandamide-stimulated cannabinoid receptor signaling in human ULTR myometrial smooth muscle cells. | 19477951 | GAPDH |  |  |  |
| Cyclic AMP increases COX-2 expression via mitogen-activated kinase in human myometrial cells. | 21854542 | GAPDH |  |  |  |
| Effects of 4-hydroxy-2-nonenal, a major lipid peroxidation-derived aldehyde, and N-acetylcysteine on the cyclooxygenase-2 expression in human uterine myometrium. | 21212636 | GAPDH |  |  |  |
| Epigenetic modulation of the protein kinase A RIIα (PRKAR2A) gene by histone deacetylases 1 and 2 in human smooth muscle cells. | 19818097 | GAPDH |  |  |  |
| Identification of a myometrial molecular profile for dystocic labor. | 21999197 | GAPDH |  |  |  |
| Influence of extracellular matrix on cytokine stimulated pro-labour gene expression in human uterine myocytes. | 19050328 | GAPDH |  |  |  |
| Melatonin synergizes with oxytocin to enhance contractility of human myometrial smooth muscle cells. | 19001515 | GAPDH |  |  |  |
| NF-κB regulates a cassette of immune/inflammatory genes in human pregnant myometrium at term. | 20406326 | GAPDH |  |  |  |
| Regulation of catechol-O-methyltransferase expression in human myometrial cells. | 17138778 | GAPDH |  |  |  |
| SK3 channel expression during pregnancy is regulated through estrogen and Sp factor-mediated transcriptional control of the KCNN3 gene. | 20682843 | GAPDH |  |  |  |
| Stretch and interleukin 1 beta: pro-labour factors with similar mitogen-activated protein kinase effects but differential patterns of transcription factor activation and gene expression. | 17348037 | GAPDH |  |  |  |
| The interleukin 1beta-induced expression of human prostaglandin F2alpha receptor messenger RNA in human myometrial-derived ULTR cells requires the transcription factor, NFkappaB. | 16855208 | GAPDH |  |  |  |
| Transcriptional inhibition of oxytocin receptor expression in human myometrial cells by melatonin involves protein kinase C signaling. | 17726073 | GAPDH |  |  |  |
| Uterine stretch and progesterone action. | 21450990 | GAPDH |  |  |  |
| Beta-Estradiol Regulates Voltage-Gated Calcium Channels and Estrogen Receptors in Telocytes from Human Myometrium. | 29747396 | GAPDH |  |  |  |
| TRPC1, STIM1, and ORAI influence signal-regulated intracellular and endoplasmic reticulum calcium dynamics in human myometrial cells. | 21565997 | GUSB |  |  |  |
| RKIP is decreased in laboring myometrium and modulates inflammation-induced pro-labor mediators. | 28280133 | LRP10 | SDHA |  | YES |
| Genes for prostaglandin synthesis, transport and inactivation are differentially expressed in human uterine tissues, and the prostaglandin F synthase AKR1B1 is induced in myometrial cells by inflammatory cytokines. | 20595240 | POLR2A | ARHGDIA |  | YES |
| Effects of medroxyprogesterone acetate on gene expression in myometrial explants from pregnant women. | 20843944 | PPIA* | B2M | GUSB | YES |
| Expression of gastrin-releasing peptide is increased by prolonged stretch of human myometrium, and antagonists of its receptor inhibit contractility. | 22411014 | PPIA* | B2M | GUSB | YES |
| A novel human cell culture model to study visceral smooth muscle phenotypic modulation in health and disease. | 30044660 | PPIA* | B2M |  |  |
| Progesterone receptor plays a major antiinflammatory role in human myometrial cells by antagonism of nuclear factor-kappaB activation of cyclooxygenase 2 expression. | 16772530 | PPIA* |  |  |  |
| HoxA13 Regulates Phenotype Regionalization of Human Pregnant Myometrium. | 26485220 | PSMD2 | RPL32 | ACTB | YES |
| Histone deacetylase inhibitors exert time-dependent effects on nuclear factor-kappaB but consistently suppress the expression of proinflammatory genes in human myometrial cells. | 18375836 | RPL19 |  |  |  |
| Oxytocin activates NF-κB-mediated inflammatory pathways in human gestational tissues. | 25451977 | RPL19 | GAPDH |  |  |
| Profiling of GABAA and GABAB receptor expression in the myometrium of the human uterus. | 30343129 | RPL27 | RPL13A |  |  |
| Tenascin-X in amniotic fluid and reproductive tissues of pregnancies complicated by infection and preterm prelabor rupture of membranes†. | 30277495 | RPL30 | B2M |  |  |
| Expression, Regulation, and Function of the Calmodulin Accessory Protein PCP4/PEP-19 in Myometrium. | 30744532 | RPLP0 |  |  | YES |
| The microRNA (miR)-199a/214 cluster mediates opposing effects of progesterone and estrogen on uterine contractility during pregnancy and labor. | 22973051 | RPLP0 |  |  |  |
| The transcriptional repressor GATAD2B mediates progesterone receptor suppression of myometrial contractile gene expression. | 28576827 | RPLP0 |  |  |  |
| Estrogen receptor alpha isoform ERdelta7 in myometrium modulates uterine quiescence during pregnancy. | 30502052 | RPLP0 |  |  |  |
| miR-200 family and targets, ZEB1 and ZEB2, modulate uterine quiescence and contractility during pregnancy and labor. | 21079000 | RPLP0 | U6 |  |  |
| A molecular signature of an arrest of descent in human parturition. | 21284969 | RPLP0 |  |  |  |
| Sodium leak channel, non-selective contributes to the leak current in human myometrial smooth muscle cells from pregnant women. | 26134120 | TOP1 | SDHA |  |  |
| Pro-inflammatory cytokine-induced microRNA-212-3p expression promotes myocyte contraction via methyl-CpG-binding protein 2: a novel mechanism for infection-related preterm parturition. | 30892651 | U6 | GAPDH |  |  |
| Identification and functional analysis of microRNA in myometrium tissue from spontaneous preterm labor. | 26722471 | U6 |  |  |  |
| A20, an essential component of the ubiquitin-editing protein complex, is a negative regulator of inflammation in human myometrium and foetal membranes. | 28911210 | YWHAZ | SDHA |  |  |
| Expression and function of macrophage-inducible C-type lectin (Mincle) in inflammation driven parturition in fetal membranes and myometrium. | 30793298 | YWHAZ | SDHA |  |  |
| IRF5 is increased in labouring myometrium and regulates pro-labour mediators. | 30006439 | YWHAZ | SDHA |  |  |
| Role of IRG1 in Regulating Pro-inflammatory and Pro-labor Mediators in Human Myometrium. | 32046417 | YWHAZ | SDHA |  |  |
| SMAD7 regulates proinflammatory and prolabor mediators in amnion and myometrium. | 29044425 | YWHAZ | SDHA |  |  |
| Delineating differential regulatory signatures of the human transcriptome in the choriodecidua and myometrium at term labor. | 29329366 | YWHAZ |  |  |  |
| Characterization of the myometrial transcriptome in women with an arrest of dilatation during labor. | 23893668 | 18S RNA | GAPDH | ACTB |  |
| Oxytocin-stimulated NFAT transcriptional activation in human myometrial cells. | 22902539 | 18S RNA | POLR2A |  |  |
| Steroid receptor co-activator interacting protein (SIP) mediates EGF-stimulated expression of the prostaglandin synthase COX2 and prostaglandin release in human myometrium. | 27161844 | 18S RNA | POLR2A |  |  |
| Circadian clock regulation of melatonin MTNR1B receptor expression in human myometrial smooth muscle cells. | 25939854 | 18S RNA |  |  | YES |
| Transcription Analysis of the Myometrium of Labouring and Non-Labouring Women. | 27176052 | 18S RNA |  |  | YES |
| A novel antiinflammatory role for the short-chain fatty acids in human labor. | 22186417 | 18S RNA |  |  |  |
| A role for lipoxin A4 as anti-inflammatory and proresolution mediator in human parturition. | 20959513 | 18S RNA |  |  |  |
| Activin-A in myometrium: characterization of the actions on myometrial cells. | 18239071 | 18S RNA |  |  |  |
| Characterization of the molecular and electrophysiological properties of the T-type calcium channel in human myometrium. | 17446221 | 18S RNA |  |  |  |
| Corticotropin-releasing hormone interacts with interleukin-1β to regulate prostaglandin H synthase-2 expression in human myometrium during pregnancy and labor. | 23666959 | 18S RNA |  |  |  |
| Diminished hERG K+ channel activity facilitates strong human labour contractions but is dysregulated in obese women. | 24937480 | 18S RNA |  |  |  |
| Effect of prolonged in vivo administration of progesterone in pregnancy on myometrial gene expression, peripheral blood leukocyte activation, and circulating steroid hormone levels. | 21558462 | 18S RNA |  |  |  |
| Effects of progesterone treatment on expression of genes involved in uterine quiescence. | 21795739 | 18S RNA |  |  |  |
| Endoplasmic reticulum stress is increased after spontaneous labor in human fetal membranes and myometrium where it regulates the expression of prolabor mediators. | 25100709 | 18S RNA |  |  |  |
| Expression and regulation of prostaglandin E synthase isoforms in human myometrium with labour. | 17105783 | 18S RNA |  |  |  |
| Expression of stretch-activated two-pore potassium channels in human myometrium in pregnancy and labor. | 20811500 | 18S RNA |  |  |  |
| Expression of the GTP-binding protein Gαs in human myometrial cells is regulated by ubiquitination and protein degradation: involvement of proteasomal inhibition by trichostatin A. | 22875848 | 18S RNA |  |  |  |
| Increased progesterone receptor A expression in labouring human myometrium is associated with decreased promoter occupancy by the histone demethylase JARID1A. | 24442343 | 18S RNA |  |  |  |
| Prokineticin 1 induces inflammatory response in human myometrium: a potential role in initiating term and preterm parturition. | 21983634 | 18S RNA |  |  |  |
| Prolonged pregnancy in women is associated with attenuated myometrial expression of progesterone receptor co-regulator Krüppel-like Factor 9. | 25313913 | 18S RNA |  |  |  |
| Term labor is associated with a core inflammatory response in human fetal membranes, myometrium, and cervix. | 19121663 | 18S RNA |  |  |  |
| Term myometrium is characterized by increased activating epigenetic modifications at the progesterone receptor-A promoter. | 22369759 | 18S RNA |  |  |  |
| The human myometrium differentially expresses mTOR signalling components before and during pregnancy: evidence for regulation by progesterone. | 23541542 | 18S RNA |  |  |  |
| The role of toll-like receptors (TLR-2 and -4) and triggering receptor expressed on myeloid cells 1 (TREM-1) in human term and preterm labor. | 19564644 | 18S RNA |  |  |  |
| The Stress-responsive Heme Oxygenase (HO)-1 Isoenzyme is Increased in Labouring Myometrium where it Regulates Contraction-associated Proteins. | 25656973 | 18S RNA |  |  |  |
| Dynamic transcriptome, accessible genome, and PGR cistrome profiles in the human myometrium. | 31908010 | 18S RNA |  |  |  |
| Exogenous oxytocin modulates human myometrial microRNAs. | 25757635 | 5S rRNA |  |  |  |
| Altered Expression of Human Smooth Muscle Myosin Phosphatase Targeting (MYPT) Isovariants with Pregnancy and Labor. | 27798640 | SM cDNA |  |  |  |
| Modulation of Progesterone Receptor Isoform Expression in Pregnant Human Myometrium. | 28540297 | ALIEN SPIKE-IN |  |  |  |
| The expression of genes involved in myometrial contractility changes during ex situ culture of pregnant human uterine smooth muscle tissue. | 28652518 | ALIEN SPIKE-IN |  |  |  |
